# Large-Scale Oral Treatment Study with the Four Most Promising D3-Derivatives for the Treatment of Alzheimer’s Disease

**DOI:** 10.3390/molecules22101693

**Published:** 2017-10-10

**Authors:** Janine Kutzsche, Sarah Schemmert, Markus Tusche, Jörg Neddens, Roland Rabl, Dagmar Jürgens, Oleksandr Brener, Antje Willuweit, Birgit Hutter-Paier, Dieter Willbold

**Affiliations:** 1 Institute of Complex Systems, Structural Biochemistry (ICS-6), Forschungszentrum Jülich, Jülich 52425, Germany; j.kutzsche@fz-juelich.de (J.K.); s.schemmert@fz-juelich.de (S.S.); m.tusche@fz-juelich.de (M.T.); d.juergens@fz-juelich.de (D.J.); 2 QPS Austria GmbH, Grambach A-8074, Austria; joerg.neddens@qps.com (J.N.); roland.rabl@qps.com (R.R.); birgit.hutter-paier@qps.com (B.H.-P.); 3 Institut für Physikalische Biologie, Heinrich-Heine-Universität Düsseldorf, Düsseldorf 40225, Germany; brener@biophys.uni-duesseldorf.de; 4 Institute of Neuroscience and Medicine, Medical Imaging Physics (INM-4), Forschungszentrum Jülich, Jülich 52425, Germany; a.willuweit@fz-juelich.de

**Keywords:** Alzheimer’s disease, amyloid β, Aβ oligomers, oral treatment, transgenic mice, d-enantiomeric peptides, large-scale, cognition

## Abstract

Alzheimer’s disease (AD) is a progressive neurodegenerative disorder that is associated with the aggregation of the amyloid β protein (Aβ). Aβ oligomers are currently thought to be the major neurotoxic agent responsible for disease development and progression. Thus, their elimination is highly desirable for therapy development. Our therapeutic approach aims at specific and direct elimination of toxic Aβ oligomers by stabilizing Aβ monomers in an aggregation-incompetent conformation. We have proven that our lead compound “D3”, an all d-enantiomeric-peptide, specifically eliminates Aβ oligomers in vitro. In vivo, D3 enhances cognition and reduces plaque load in several transgenic AD mouse models. Here, we performed a large-scale oral proof of concept efficacy study, in which we directly compared four of the most promising D3-derivatives in transgenic mice expressing human amyloid precursor protein with Swedish and London mutations (APP_SL_), transgenic mice, to identify the most effective compound. RD2 and D3D3, both derived from D3 by rational design, were discovered to be the most effective derivatives in improving cognition in the Morris water maze. The performance of RD2- and D3D3-treated mice within the Morris water maze was significantly better than placebo-treated mice and, importantly, nearly as good as those of non-transgenic littermates, suggesting a complete reversal of the cognitive deficit of APP_SL_ mice.

## 1. Introduction

Alzheimer’s disease (AD) is known to be the most common form of dementia worldwide. Although the detailed reasons for the development of the disease are still not fully understood, major pathological hallmarks of this disease are extracellular amyloid β protein (Aβ) accumulations (plaques), neurofibrillary tangles of the hyperphosphorylated protein tau, neuroinflammation, and neurodegeneration, all together resulting in cognitive decline of the affected patients. Over decades, it was thought that insoluble Aβ plaques are the main cause of cognitive decline. Nowadays, soluble Aβ oligomers are suggested to be the main toxic agent that is responsible for the development and progression of AD [1,2]. Therefore, elimination of those toxic Aβ oligomers appears to be a promising strategy for finding a curative and disease modifying therapy against AD [2,3]. Antibodies that claim to be specific for Aβ oligomers may potentially bind them, without directly destroying them and without relying on the immune system or parts thereof.

Within our group, we focus on a therapeutic strategy to specifically and directly eliminate toxic Aβ oligomers, by ligand-mediated stabilisation of Aβ monomers in an aggregation-incompetent conformation. This is supposed to shift any equilibrium between Aβ species towards Aβ monomers and away from toxic Aβ oligomers. For this purpose, we developed different peptides that solely consist of d-enantiomeric amino acid residues (d-peptides), by mirror image phage display [4,5]. Based on this, our lead compound “D3” arose. In various studies, it has been proven that D3 specifically eliminates Aβ oligomers in vitro, inhibits Aβ fibril formation, and reduces Aβ-mediated cytotoxicity. In vivo, D3 is able to reduce plaque load and enhance cognition in several transgenic AD mouse models, even after oral administration [6,7,8,9]. With the intention to improve the oligomer-eliminating efficacy of D3 in vitro, we systematically identified different derivatives of D3 [10,11,12]. In addition, we pursued a different approach by rationally designing new D3-derivatives to increase knowledge in regard to essential sequence motifs and to increase efficacy. In this context, we identified four compounds with very promising in vitro properties, especially regarding their efficiency in Aβ oligomer elimination. Those include “RD2”, which consists of exactly the same amino acid residues as D3 but in a re-arranged sequence; “D3D3” and “RD2RD2”, the head-to-tail linear connected homodimers of the lead compounds D3 and RD2, respectively; as well as the head-to-tail linear connected heterodimer of RD2 and D3, termed “RD2D3”. In vitro, all these newly designed d-peptides were able to eliminate toxic Aβ oligomers more efficiently than the lead compound D3 [8]. For D3D3, it could be additionally shown that it improved cognitive performance of transgenic APP-Swedish, Dutch, Iowa (Tg-SwDI) mice and reduced Aβ plaque load within the hippocampus and cortex [8]. Pharmacokinetic studies—which were performed with D3, RD2, D3D3, and RD2D3—have shown that all compounds efficiently cross the blood–brain barrier with brain–plasma ratios close to one after three to six hours after application and long half-lives in plasma and brain [13,14,15].

In the current report, we performed a large-scale oral proof of concept study with a total of 88 mice, in which we directly compared the efficacy of the four most promising D3-derivatives—RD2, RD2RD2, RD2D3, and D3D3—in the transgenic mouse model APP-Swedish, London (APP_SL_). RD2 and D3D3 were found to be the most effective derivatives based on the significantly improved cognitive performance in the Morris water maze (MWM) compared to placebo-treated mice. Most importantly, the cognitive deficit of APP_SL_ mice was completely reversed and learning abilities of both treatment groups were similar to those of non-transgenic littermates.

## 2. Results

### 2.1. D3-Derivatives Eliminated Aβ Oligomers in the QIAD Assay and Significantly Reduced Aβ-Induced Cell Toxicity

**Figure 1:**
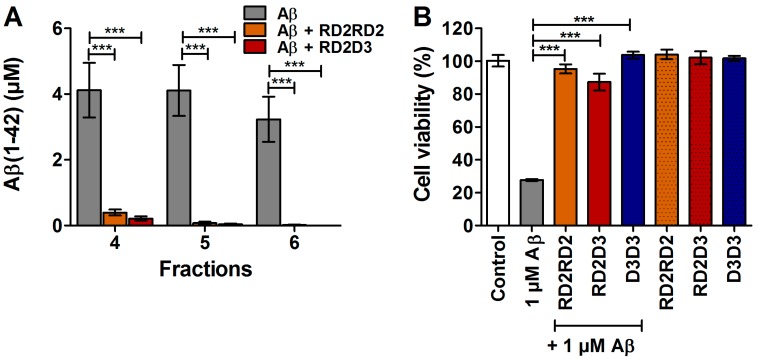
RD2RD2 and RD2D3 eliminated toxic amyloid β protein (Aβ) oligomers and reduced Aβ induced cell toxicity. Potency of RD2RD2 and RD2D3 to reduce toxic Aβ oligomers was demonstrated in vitro by the QIAD assay (**A**). Both compounds were able to reduce the toxic Aβ oligomers present in fractions 4–6. Cell viability was assessed by MTT (3-(4,5-dimethylthiazol-2-yl)-2,5-diphenyl-tetrazolium bromide) after incubation of PC-12 cells (**B**) with 1 µM Aβ(1–42) co-incubated with 1 µM RD2RD2 (orange), 1 µM RD2D3 (red), or 1 µM D3D3 (blue). All compounds significantly increase the cell viability after co-incubation with Aβ(1–42), without having negative impact on cell viability when incubated alone (patterned bars). Data is represented as mean ± SD (standard deviation), one-way ANOVA (analysis of variance) with Fisher post hoc analysis, *** *p* ≤ 0.001.

Previously, we have already proven that RD2 and D3D3 were able to eliminate Aβ(1–42) oligomers significantly more efficiently than the lead compound D3 [8,16]. In this study, the potency of the D3-derivatives RD2RD2 and RD2D3 regarding their Aβ oligomer eliminating abilities was investigated in vitro by performance of a quantitative determination of interference with the Aβ aggregate size distribution (Aβ QIAD) assay. Findings of this analysis demonstrate that RD2RD2 and RD2D3 also significantly and efficiently eliminated toxic Aβ(1–42) oligomers (Figure 1A).

Furthermore, the outcome of the 3-(4,5-dimethylthiazol-2-yl)-2,5-diphenyl-tetrazolium bromide (MTT) cell viability assay with PC-12 cells revealed that all compounds were capable of significantly reversing the cellular toxicity exerted by 1 µM preincubated Aβ(1–42) (Figure 1B and [7,16]), without negative impact on cell viability on their own.

### 2.2. RD2 and D3D3 Treated Mice Exhibited Improved Cognitive Performance

Oral treatment of 75 APP_SL_ mice with either placebo, RD2, RD2RD2, RD2D3, or D3D3 did not result in significant gain or loss of body weight before vs. after treatment or in any change of general conditions of the mice compared to placebo-treated mice or non-transgenic littermates (ntg) (body weight before vs. after treatment: placebo 23.8 g vs. 23.8 g; RD2 23.5 g vs. 23.7 g; RD2RD2 23.9 g vs. 23.7 g; RD2D3 24.6 g vs. 23.8 g; RD2D3 24.7 g vs. 24.5 g; ntg 25.6 g vs. 25.7 g).

**Figure 2:**
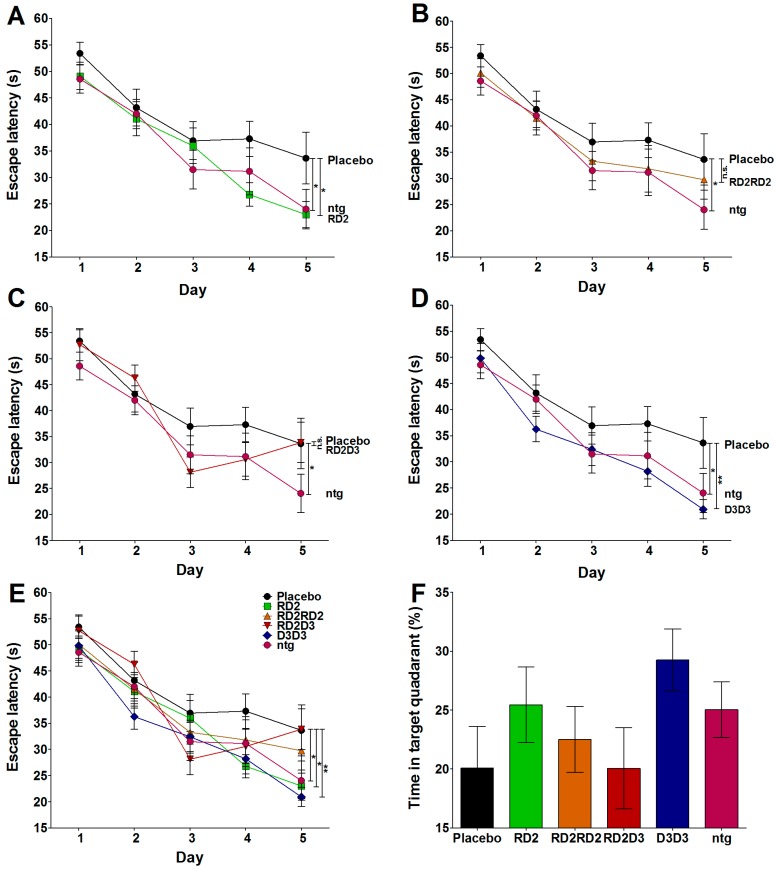
Treatment with RD2 and D3D3 improved cognitive performance as shown in the Morris water maze (MWM). During the acquisition phase of the MWM, RD2- (green, (**A**,**E**)) and D3D3- (blue, (**D**,**E**)) treated mice showed significantly improved learning behaviors compared to those of placebo- (black) treated mice (two-way repeated measurements (RM) ANOVA, Fisher post hoc analysis, * *p* < 0.05, ** *p* < 0.001) (**A**–**C**,**E**). The improved cognitive performance is indistinguishable to that of non-transgenic littermates (ntg, pink) ((**A**–**C**)). Neither RD2RD2- (orange (**B**,**E**)) nor RD2D3- (red, (**C**,**E**)) treated mice showed improved cognitive performance compared to the behavior of placebo-treated mice. (**E**) is a combination of (**A**) to (**D**) for direct comparison of all four tested compounds. The probe trial did not reveal significant differences between the groups, but showed the tendency of RD2- and D3D3-treated mice to spend more time in the target quadrant **F**.

The Morris water maze (MWM) was performed to explore whether treatment with the compounds RD2, RD2RD2, RD2D3, or D3D3 results in cognitive improvement. The results were compared to the performance of placebo-treated mice and non-transgenic littermates. As demonstrated in Figure 2, treatment with RD2 (Figure 2B) and D3D3 (Figure 2C) achieved a significant improvement of cognition during the acquisition phase of the MWM (two-way RM ANOVA, Fisher post hoc analysis: placebo vs. ntg *p* = 0.039, placebo vs. RD2 *p* = 0.02, placebo vs. D3D3 *p* = 0.006), which was similar to those of non-transgenic littermates. Treatment with RD2RD2, the head-to-tail tandem compound of RD2, did not show a significant improvement in cognition, nor did RD2D3 (two-way RM ANOVA, Fisher post hoc analysis: placebo vs. RD2RD2 or RD2D3 non-significant (n.s.)), a combination of the compounds RD2 and D3. There was no significant difference determined between the swimming speed and the covered path length of all analyzed groups.

The probe trial testing memory-retrieval did not reveal significant differences between the tested groups (one-way ANOVA, n.s. all groups, Figure 2D), although there is a clear tendency of RD2- and D3D3-treated mice just as non-transgenic littermates for abidance in the target quadrant.

### 2.3. RD2 and D3D3 Improved Cognitive Performance without Influencing Aβ Pathology or Inflammation

**Figure 3:**
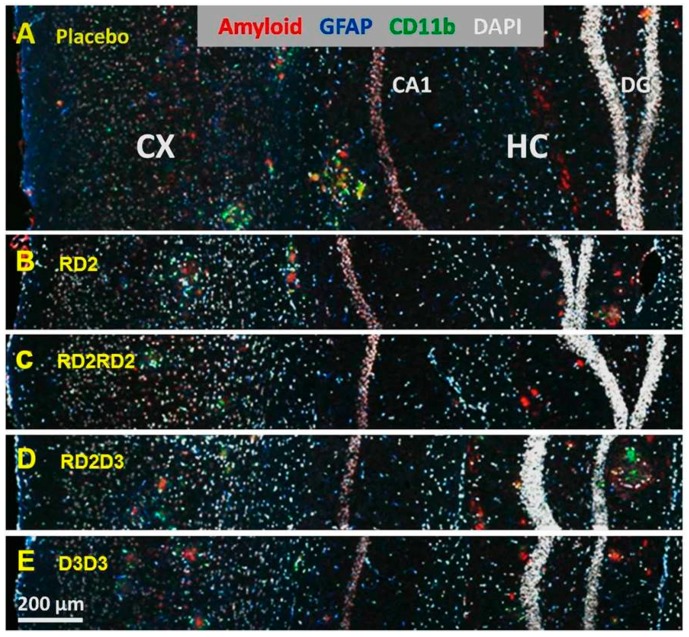
Immunofluorescent labeling of amyloid and glia cells. The images show exemplary immunofluorescent labeling of amyloid (antibody 6E10, red), astrocytes (GFAP (glial fibrillary acidic protein), blue), and activated microglia (CD11b, green) in mice of all groups (**A**–**E**). Nuclei are labeled with DAPI (white) to provide the requested comparison between the different groups. Abbreviations: CX, Cortex; CA1, *cornu Ammonis* area 1; HC, hippocampus; DG, dentate gyrus.

To investigate whether treatment with the four D3-derivatives has any influence on Aβ deposits or neuroinflammation in APP_SL_ transgenic mice, different staining procedures on histological brain sections were performed (Figure 3) and stained areas were analyzed within the hippocampus and cortex. Staining with 6E10 antibody against transgenic human Aβ was performed to visualize Aβ deposits in the hippocampus and cortex. There were no significant differences in the stained area in both regions analyzed of all treated groups compared to placebo-treated mice, but there was a tendency of lowering Aβ deposits within the cortex by treatment with RD2 (6E10 IR area (%): RD2 7.4 ± 0.4 vs. placebo 8.4 ± 0.6, one-way ANOVA, *p* = 0.22).

By analysis of two populations of glia cells—glial fibrillary acidic protein (GFAP)-expressing astrocytes and CD11b-expressing reactive microglia—no significant effect of RD2, RD2RD2, RD2D3, or D3D3 treatment was achieved compared to placebo-treated mice, suggesting that there is no change within the neuroinflammatory status of the treated mice (Figure 3A–E).

## 3. Discussion

One of the major challenges of the current century is finding a curative and disease-modifying treatment for Alzheimer’s disease, a devastating neurodegenerative disorder. Despite intensive research, approved medications are by now not able to slow down disease progression, but are only able to treat symptoms in a very limited way, often combined with unattractive side effects. Over the past years, we developed all-d-enantiomeric peptides for direct and specific elimination of toxic Aβ oligomers as an attractive treatment strategy for AD. The lead compound D3 specifically eliminates toxic Aβ oligomers in vitro [8]. Eight weeks of oral treatment of four-months-old APP/PS1 mice with moderate doses of D3 significantly improved cognition compared to untreated mice, as tested in the Morris water maze. Additionally, Aβ plaque load in hippocampus and frontal cortex, as well as plaque-related inflammation, was significantly reduced in D3-treated versus untreated mice [9]. In the present study, we compared in vivo efficiencies of the D3-derivatives RD2, RD2RD2, RD2D3, and D3D3, which were the most efficient compounds in vitro. The direct comparison of the influence of RD2 vs. D3 or D3D3 vs. D3 on Aβ oligomer elimination efficiencies, as measured by the QIAD assay, revealed that all tested peptides significantly reduced the amount of Aβ oligomers; however, RD2 and D3D3 performed significantly better than D3 (RD2 71%, D3D3 96%, and D3 51% oligomer reduction) [8,16]. In vivo in the current study, it was demonstrated that RD2 and D3D3 improved cognitive performances of APP_SL_ mice by oral application of moderate doses over six weeks. The hereby described comparison is based on the same single dose for each compound. Dose–response curves for the most promising compounds might give an even more quantitative basis for future comparisons. The cognitive behavior of both treated groups was similar to the behavior of non-transgenic littermates, suggesting a reversal of the cognitive impaired phenotype. In contrast, RD2RD2 and RD2D3 did not yield significant improvement of cognitive performance within this study, as neither compound changed the amyloid load or neuroinflammatory pathology within the therapeutic time frame.

The finding that cognitive improvement was achieved by treatment with RD2 and D3D3 without significantly changing the typical plaque pathology underlines the hypothesis that plaque pathology is not directly correlated to cognitive deficits. Thus, more and more evidence is given that, instead of Aβ assemblies in senile plaques, soluble Aβ oligomers are the main cause of cognitive decline [17,18,19,20]. Also, in patients, several studies demonstrated that Aβ plaque load is not directly correlated with cognitive decline [21,22].

Based on the in vitro findings, we suggest for our compounds the specific and direct elimination of toxic oligomers by stabilizing Aβ monomers in an aggregation-incompetent conformation as a possible mode of action. This shifts any equilibrium between Aβ species towards Aβ monomers and away from Aβ oligomers, leading to the elimination of toxic Aβ oligomers [8,9]. Taking advantage of this strategy, our compounds do not recognize any distinct Aβ oligomer species, thus avoiding any danger of resistance development. In addition, they not only readily prevent Aβ oligomer formation but also eliminate oligomers that are already formed. We conjecture, sustained by our in vitro results, that this mode of action eventuates in vivo, too. Within this study, a reversal of cognitive deficits by treatment with RD2 and D3D3, most likely by a specific elimination of toxic Aβ oligomers and without reducing senile plaques, was demonstrated. As described in the literature [1], the reduction of Aβ oligomers results in improved synaptic function, resulting in improved cognitive performance. Irrespective of that, the observations made in this study strengthen the hypothesis that there is no crucial link between plaque pathology and cognition. Based on our hypothesis of shifted Aβ equilibrium by treatment with our compounds, long-term therapy of several months may casually also result in reduced plaque load, as previously demonstrated for D3 [7].

In conclusion, RD2 and D3D3 were both able to significantly enhance the cognitive abilities of APP_SL_ mice. In consequence, both are predicted to be promising drug candidates for anti-amyloid treatment. Nevertheless, findings of pharmacokinetic studies, conducted with RD2 and D3D3, indicate that RD2 has more favorable properties in comparison to D3D3, especially concerning the terminal half-life and oral bioavailability [14,15], so that an accurate therapeutic regimen with once daily administration is feasible. This is of great advantage to enable a suitable compliance of orally administered drugs, especially for a drug intended to be given to patients with dementia.

## 4. Materials and Methods

### 4.1. Ethics Statement

All animal experiments conformed to the Austrian guidelines for the care and use of laboratory animals and were approved by the Styrian Government, Amt der Steiermärkischen Landesregierung Abteilung 13-Umwelt und Raumordnung, Austria (Project ID: ABT13-78Jo142-2014, date of approval: 18 June 2014).

### 4.2. Peptides

All peptides—RD2, RD2RD2, RD2D3, and D3D3—were C-terminally amidated and purchased from Cambridge Peptides (Cambridge Peptides, Birmingham, UK) and Peptides and Elephants (Peptides & Elephants, Henningsdorf, Germany) as lyophilized powder with a minimal purity of 97% (Table 1).

### 4.3. QIAD Assay

To investigate the Aβ oligomer elimination properties of RD2RD2 and RD2D3 in vitro, a QIAD assay (quantitative determination of interference with Aβ aggregate size distribution) was performed according to Brener et al. [6]. In short, 10 µM of the D3-derivatives RD2RD2 or RD2D3 were added to pre-incubated 80 µM Aβ(1–42) solution. Afterwards, samples were fractionated on a 5–50% (*w*/*v*) iodixanol gradient (OptiPrep, Axis-Shield, Norway) and ultracentrifuged for 3 h at 4 °C and 259.000× g (Optima TL-100, Beckman Coulter, Brea, CA, USA). Subsequently, the resulting 14 fractions, each 140 µL, were taken top to bottom from the gradient. Obtainment of the 15th fraction was performed by boiling the pellet for 10 min after mixing with 60 µL of 6 M guanidine hydrochloride. The oligomer containing fractions 4 to 6 was analyzed by analytical reversed phase-high performance liquid chromatography (RP-HPLC) and ultraviolet absorbance was detected at 214 nm. The Aβ oligomer elimination properties of RD2 and D3D3 were previously published [8,16].

### 4.4. Cell Viability Test

Examination of Aβ(1–42)-induced cell toxicity in the presence or absence of the D3-derivatives RD2RD2, RD2D3, and D3D3 was conducted by use of a MTT-based cell viability assay as described previously (e.g., [11,12]). For this purpose, PC-12 phaeochromocytoma cells (DSMZ, Braunschweig, Germany) were cultured in DMEM medium supplemented with 10% fetal calf serum, 1% antibiotics (Penicillin/Streptomycin) (all Sigma-Aldrich, St. Louis, MO, USA), and 5% horse serum (PAA Laboratories GmbH, Cölbe, Germany) on collagen A-coated (Biochrom GmbH, Berlin, Germany) tissue culture flasks (SPL Life Sciences Co., Naechon-Myeon, Korea). Cells were allowed to grow in a humidified incubator with 5% CO_2_ at 37 °C and a maximum of 12 passages, whereby the medium was changed every two days. Cells were passaged according to their confluence.

In accordance with the manufacturer’s protocol, an MTT test was performed (CellProliferation Kit I; Roche, Basel, Switzerland). After the cells were seeded in clear, collagen-coated 96-well flat bottom microwell plates (Life Technologies Inc., Carlsbad, CA, USA) in a volume of 100 μL per well at a density of 1 × 10^4^ cells, they were incubated for 24 h. On the next day, compounds were prepared in the following manner: after incubation of 51 µM Aβ(1-42) for 4.5 h at 37 °C shaking at 600 rpm, 25 µM RD2RD2, RD2D3, or D3D3 were added and incubated for another 40 min. During the test, cells were exposed to 1 µM Aβ with or without 0.5 µM RD2RD2, RD2D3, or D3D3. All experiments were conducted as a fivefold determination in three independent experiments. The arithmetic mean of all measurements per approach was calculated. Data is represented as the percentage of MTT reduction, assuming that the absorbance of control cells was 100%.

### 4.5. Mice

Female 8 months ± 2 weeks-old APP_SL_ mice were used in the present study. Mice were bred hemizygous for the transgene on a C57BL/6 background. Behavioral and pathological characteristics of the used mouse model are previously described [23,24]. Mice were group-housed in individually ventilated cages on standard rodent bedding (Rosenberg, Germany) in the AAALAC-accredited animal facility of QPS Austria GmbH, Grambach, Austria) with a 12/12 h light dark cycle, 21 °C room temperature, 40–70% humidity and food and water ad libitum. Only mice in good health condition were included in the study. Randomization of group allocation was done per cage. All mice of a cage were randomly assigned to a treatment group. Mice were assigned to different starting groups (cohorts) comprising mice of all treatment groups. The number of mice in a starting group was limited to ensure same age and uniform handling.

### 4.6. Treatment

Female APP_SL_ mice, aged 32 ± 2 weeks, were orally treated once daily for six weeks. Administration was performed by gavage with 200 μL of test compound diluted in 0.9% NaCl or placebo with 20 mg/kg of either RD2 (*n* = 15), RD2RD2 (*n* = 15), RD2D3 (*n* = 15), D3D3 (*n* = 14), or 0.9% NaCl as control group (placebo). The administered dose in this study (20 mg/kg) was derived from a previous D3 treatment study [9]. In this study, mice with a body weight of approximately 25 g were orally treated with 0.5–1 mg D3 daily. This corresponds to a daily administered dose of 20 mg/kg. The administered doses were derived from the therapeutic efficient dosage from the first oral D3 treatment study [9]. For the validation of the Morris water maze, a group of non-transgenic littermates was used as control. In total, 74 transgenic and 14 non-transgenic mice were used in the present study.

### 4.7. Morris Water Maze

The MWM was performed to assess spatial learning and memory performance of all groups described above. The used water maze consisted of a circular pool (diameter 100 cm) filled with opaque made water (24 ± 1°C) to a depth of 20 cm, with a hidden but fixed platform, located 0.5 cm beneath the water surface. During all trials, the platform was located in the target quadrant (northeast, NE) of the pool. The used protocol was adopted and modified from Morris et al., 1982 [25]. The acquisition phase of the MWM was performed over five consecutive days. Mice had to swim on each day four trails with a maximum length of 60 s from predefined positions. If the mice did not find the hidden platform within the 60 s, they were guided to the target and were allowed to orientate themselves for 10–15 s. The escape latency was analyzed in s, the time mice needed to escape on the hidden platform. On the sixth day, 24 h after the last day of the acquisition phase, the probe trial was performed. Thereby, the platform was removed from the pool and all mice had to swim for 60 s. The abidance of mice in the target quadrant was analyzed. Mice were observed with a camera driven tracking system, Viewer III (Biobserve GmbH, Bonn, Germany).

### 4.8. Tissue Collection

After the behavioral assessments, the mice were sacrificed to obtain brain samples. The mice were anesthetized intraperitoneally by sodium pentobarbital injection (600 mg/kg). When the mice reached the appropriate anesthesia stage, they were transcardially perfused with 0.9% saline. Subsequently, the brains were removed and the left hemispheres were frozen in dry ice and stored at −80 °C. The right hemispheres were used for immunohistochemical analyses: hemispheres were fixed in freshly prepared 4% paraformaldehyde in phosphate buffer (pH 7.4) for one h at room temperature (RT). Afterwards, hemispheres were transferred into a 15% sucrose solution for cryoprotection. On the next day, the fixed hemispheres were frozen in isopentane and stored at −80 °C until further processing.

### 4.9. Immunohistology

Immunohistochemical analysis was performed with six animals per treatment group. Sagittal cryosections (10 µm) were generated on a Leica CM 1950 cryostat (Leica Biosystems, Nussloch, Germany). In brief, five cyrosections per mouse were allowed to air-dry for 45 min at RT and washed with phosphate buffered saline (PBS) for 10 min. After incubation in sodium borohydrate solution for 4 min, cyrosections were washed two times with PBS for 10 min, permeabilized with 1% TritonX-100 in PBS for 10 min, and again washed three times in PBS for 5 min. Subsequently, after blocking of unspecific bindings with 10% horse serum in PBS for 30 min at RT, cryosections were incubated with CD11b antibody (Bio-Rad Laboratories, Puchheim, Germany; dilution 1:1000) for 60 min at RT in a humid chamber. After another washing step in PBS, cyrosections were incubated with donkey anti-rat DyLight650 (Abcam, Cambridge, UK; dilution 1:500) for 60 min at RT in a humid chamber in the dark. Following a further washing step and blocking of unspecific bindings with a mouse-on-mouse (MOM) blocking reagent (MOM-kit, Vector, Burlingame, CA, USA) for 30 min at RT in the dark, cryosections were incubated for 60 min with GFAP (Dako, Agilent technologies, Santa Clara, CA, USA; dilution 1:500) and 6E10 (BioLegend, San Diego, CA, USA; dilution 1:1000) for 30 min at RT in a light protected humid chamber. Subsequently, cryosections were incubate with donkey anti-rabbit DyLight488 (Abcam, dilution 1:500) and donkey anti-mouse AlexaFluor555 (Abcam, dilution 1:500) for 60 min at RT in a light protected humid chamber. After the washing step in PBS, cryosections were incubated with 4′,6-diamidino-2-phenylindole for 15 min and differentiated two times with 80% ethanol for 2 min. Cryosections were mounted and cover-slipped. All sections were stained in equal volumes to avoid differences in staining intensity.

### 4.10. Quantification

All sections were acquired in one microscopy session to avoid changes in light exposure, which might affect measurements. Visualization of sections was conducted by use of a Zeiss Axio Imager Z1 microscope (Zeiss, Jena, Germany) with a high aperture lens (10× lens, numerical aperture 0.90, 1× optocoupler), equipped with a Zeiss AxioCam MRm camera and Zeiss AxioVision 4.8 software (Zeiss, Jena, Germany). Quantification was performed with ImageProPlus software (Media Cybernetics, Inc., Rockville, MD, USA).

### 4.11. Statistics

All statistical calculations were conducted by SigmaPlot Version 11 (Systat Software, Erkrath, Germany). Data is represented as mean ± SD (in vitro results) or SEM (in vivo results). Data was analyzed using one-way ANOVA with Fisher LSD post hoc analysis. Repeated measurements (RM) were analyzed by use of two-way RM ANOVA with Fisher LSD post hoc analysis. Significant results of the MWM were confirmed by use of Friedman Repeated Measures ANOVA on Ranks.

## Figures and Tables

**Table 1:** Amino acid sequences of the applied d-enantiomeric peptides.

Peptide	Sequence (All d-Enantiomeric)
RD2	ptlhthnrrrrr
RD2RD2	ptlhthnrrrrrptlhthnrrrrr
RD2D3	ptlhthnrrrrrrprtrlhthrnr
D3D3	rprtrlhthrnrrprtrlhthrnr
